# Exploring the Critical Thickness of Organic Semiconductor Layer for Enhanced Piezoresistive Sensitivity in Field-Effect Transistor Sensors

**DOI:** 10.3390/ma13071583

**Published:** 2020-03-30

**Authors:** Damien Thuau, Katherine Begley, Rishat Dilmurat, Abduleziz Ablat, Guillaume Wantz, Cédric Ayela, Mamatimin Abbas

**Affiliations:** 1 Laboratoire IMS, Université de Bordeaux, CNRS, Bordeaux INP, UMR 5218, F-33607 Pessac, France; Katherine.Begley@ims-bordeaux.fr (K.B.); rishat.dilmurat@ims-bordeaux.fr (R.D.); abulaiti.abuduaizezi@u-bordeaux.fr (A.A.); guillaume.wantz@ims-bordeaux.fr (G.W.); cedric.ayela@ims-bordeaux.fr (C.A.); 2 Institut Universitaire de France (IUF), 75005 Paris, France

**Keywords:** organic semiconductor, organic field-effect transistor, piezoresistivity, sensors

## Abstract

Organic semiconductors (OSCs) are promising transducer materials when applied in organic field-effect transistors (OFETs) taking advantage of their electrical properties which highly depend on the morphology of the semiconducting film. In this work, the effects of OSC thickness (ranging from 5 to 15 nm) on the piezoresistive sensitivity of a high-performance p-type organic semiconductor, namely dinaphtho [2,3-b:2,3-f] thieno [3,2–b] thiophene (DNTT), were investigated. Critical thickness of 6 nm thin film DNTT, thickness corresponding to the appearance of charge carrier percolation paths in the material, was demonstrated to be highly sensitive to mechanical strain. Gauge factors (GFs) of 42 ± 5 and −31 ± 6 were measured from the variation of output currents of 6 nm thick DNTT-based OFETs engineered on top of polymer cantilevers in response to compressive and tensile strain, respectively. The relationship between the morphologies of the different thin films and their corresponding piezoresistive sensitivities was discussed.

The phenomenon of piezoresistivity dates back to 1954 when Smith et al. reported the piezoresistive effect in germanium and silicon [1]. Since then, the piezoresistivity of inorganic semiconductors has been extensively studied [2]. Over the last years, the microelectronic industry is increasingly considering the use of organic semiconductors. The idea is to substitute their inorganic counterparts partially or completely in various electronic devices, i.e., organic light-emitting diodes (OLEDs) [3,4], organic photodetector (OPD) [5] and organic field-effect transistors (OFETs) [6]. Regarding the latter, OFETs offer great potential for flexible electronic applications such as flexible displays [7], memories [8] and sensors [9]. OFET-based sensors use π-conjugated organic polymers or small molecules as active materials whose electronic properties, notably their charge carrier transport, can be influenced by physical, chemical and biological stimuli [9,10]. Various approaches were investigated to enhance the performance of OFET-based sensors. Wu et. al. reviewed the most popular routes that consisted of the modification of the OSC molecular structures, micro/nano structuration of the OSC or electrodes and an unconventional choice of a pair of OSC/dielectric materials to induce interfacial charge traps [11]. To date, most of the studies reporting efficient OFET sensors relied on capacitive detection [12,13]. On the contrary, mechanical strain effects on the electrical properties of OSC-based sensors have not been reported with thorough investigations and mostly concerned pentacene as an active layer material, which is known to be unstable in ambient conditions [14,15]. Therefore, an evaluation and optimization of mechanical strain effects on the electrical properties of emerging, stable OSCs needs to be addressed to strengthen their uses in either strain sensitivity (sensors) or strain stability (flexible electronics) oriented applications. Dinaphtho [2–b:2,3–f] thieno [3,2–b] thiophene (DNTT) belongs to the well-known class of acene-based organic semiconductors [16,17]. Vacuum evaporated DNTT exhibits both high carrier mobility of over 1 cm²∙V^–1^∙s^–1^ and excellent air stability. Reeder et al. investigated the strain stability of DNTT-based OFET sensors for implantable electronics. While charge carrier mobility was observed to vary by only 0.7% under an applied compressive strain of 0.8%, significantly larger on-current changes were measured (~20%). [18] To enhance the strain sensitivity of DNTT OFET sensors, we previously introduced an active piezoelectric gate dielectric polymer on the DNTT-based OFET sensors [19]. The enhancement of the sensitivity of the piezoelectric gated OFET by a factor 18 originated from the piezoelectric material itself, which affects the electrical behavior of the transistor as a signature of a mechanical event. Recently, Wang et al. also reported a highly sensitive piezoresistive (514 kPa^−1^) sensor made of DNTT-based OFET [20]. In their case, the piezoresistive effect came from the semiconductor/conductor contact interface where Au contact electrodes were micro-structured into pyramid shapes to improve the detection sensitivity. To date, the influence of the thickness of the semiconductor, i.e., tuning the coverage of the OSC on the dielectric, has never been investigated in the framework of piezoresistive OFET force sensors. In this work, we explore the critical thickness of the active layer for maximizing the piezoresistive sensitivity of an OFET sensor. The strain sensitivity of the OFET sensor is found to be significantly enhanced by adjusting the thickness of the DNTT layer close to the percolation threshold of the active layer (~6 nm).

The device architecture is a CantiFET, an OFET embedded in a suspended organic micro-cantilever, which was previously reported [19,21]. The OFETs have a bottom gate/top contact structure fabricated on flexible 50 μm thick polyethylene naphthalate (PEN) substrates (Figure 1a). PEN substrates were cleaned in acetone, isopropanol and deionized water baths successively for 10 minutes. An electrode of a thick (100 nm) Aluminum (Al) film was deposited and patterned by e-beam evaporation at low pressure (5 × 10^−7^ mbar) on the substrates through shadow masks as gate electrodes. The Al gate was partially anodized to obtain 35 nm of Al_2_O_3_ dielectric. Polystyrene (PS) (700,000 g.mol^−1^) was dissolved in chlorobenzene with a 3 mg∙mL^−1^ concentration. PS solution was then spin-coated at 2000 rpm for 60 s in order to form very thin passivation layers on the Al_2_O_3_ surface and dried in a vacuum oven at 80 °C for two hours. A metal–insulator–metal (MIM) structure, such as Al/oxide/PS/Al, was prepared to measure a total capacitance of 95 nF∙cm^−2^ of the dielectric bilayer at 10 Hz using an Agilent network analyzer. Subsequently, DNTT was evaporated at a base pressure of 1 × 10^−6^ mbar; the film thickness and the deposition rate (0.6 nm∙min^−1^) were monitored by a quartz crystal microbalance placed next to the sample. Gold (Au) was chosen as the metal for Source–Drain contacts and patterned by thermal evaporation through a shadow mask (channel length is 100 µm, while channel width is 3 mm). CantiFET devices were then obtained by xurography using a computer numerical control vinyl cutter (CAMEO Silhouette 2). The triangular shape of the cantilever was chosen to induce uniform longitudinal stress in the DNTT thin film when a force was applied at the free end of the cantilever. The thickness of the DNTT layers investigated in this work ranges from 5 nm to 30 nm. Above 15 nm thick film, no significant changes in the transfer curves of the measured OFETs were observed. A 5 nm thick DNTT film was found to be not enough to allow charge carrier transport and hence turn on the OFET. Consequently, Figure 1b compares the transfer characteristics of 6, 8 and 15 nm thick DNTT-based OFET in pristine conditions (without strain) in which the drain current, *I_DS_* (solid line), and the gate current, *I_GS_* (dashed line), as a function of the gate voltage *V_GS_* were measured at a speed of 833 mV∙s^−1^ using a Keithley 4200 semiconductor analyzer in air at room temperature. *I_DS_* was observed to decrease as the thickness of the active layer was reduced from 15 to 6 nm due to the hindered charge carrier transport along the transistor channel. We found that on-state *I_DS_* values of OFETs with OSC thicker than 15 nm did not vary as charge carrier transport takes place in the first few nanometers next to the dielectric interface provided that complete coverage is attained. This is in agreement with previously reported work on spatially correlated charge transport in organic thin film transistors [22,23]. In fact, 6 nm was found to be the minimum thickness to allow charge transport in the OSC layer, which we determined as the critical thickness, where the percolation path was achieved between DNTT grains. Additionally, decreasing the active layer thickness also increased the morphological defects, thus charge trapping sites and consequently slightly shifted the threshold voltage (*V_th_*) from −1.6 V for a 15 nm thick DNTT layer towards higher *V_GS_* at −2.2 V for 8 and 6 nm thick film as shown in Figure 1b. The hole mobility (*µ*) in saturation regime was extracted using the following Equation [24]:

$$µ=\frac{2L}{WC_{s}}{\left(\frac{\partial \sqrt{I_{DS}}}{\partial V_{GS}}\right)}^{2}$$

where *L* and *W* are the channel length and width and *C_s_* is the capacitance of the bilayer dielectric oxide/PS. We extracted hole mobility for the 15, 8 and 6 nm thick DNTT films as high as 2.45 ± 0.32 cm²∙V^−1^∙s^−1^, 0.46 ± 0.15 cm²∙V^−1^∙s^−1^ and 0.30 ± 0.18 cm²∙V^−1^∙s^−1^, respectively.

By bending the flexible cantilever, controlled and uniform strains can be applied to the embedded transistors. To do so, a 400B Force Transducer System from Aurora Scientific was employed to apply force upwards or downwards on the tip end of the sensors resulting in compressive and tensile strain, respectively, to the CantiFETs. The corresponding strain on the top surface was then calculated as *ε = hδ /L²*, where h and L are, respectively, the thickness and the length of the cantilever. The deflection (*δ*) was obtained by measuring the position of the cantilever extremity. Figure 2a shows the changes of *I_DS_* with *V_GS_* ranging from 0 to −5 V with a step of −1 V on an 8 nm thick DNTT-based OFET sensor under 0.4% of tensile strain in air at room temperature. The drain current changed significantly in response to the applied force, which can be explained by the modification under strain, of the electrical charge transport within the thin film DNTT active layer. In such an ultrathin active layer, charge carrier transport is mainly limited by the grain boundaries, which is inversely proportional to the distances between the grains. Under tensile strain, the mechanical deformation increases the distance between grains (Figure 2b), which consequently decreases the output current of the transistor. Importantly, the variation of the on-current upon the application of tensile strain is reversible and reproducible, demonstrating that the devices are still operating fine after five cycles and that the current variation is not caused by drift. Inversely, compressive strain compacts the grains decreasing the distances between them and favoring additional contacts, thus increasing the output current as shown in Figure 2c.

In order to evaluate the piezoresistive sensitivity of DNTT-based OFET strain sensors, we used the most common figure of merit of piezoresistive sensors, their gauge factor (GF). The GF is generally defined as the normalized change of resistance per applied strain, GF = (*ΔR*/*R_0_)*/*ε*, where *ΔR* is the change in resistance compared to that of the unstrained device, *R_0_* is the resistance of the unstrained device, and ε is the applied strain. In the case of the OFET-based strain sensor, the GF can be defined as the normalized drain current change per applied strain, GF = (*ΔI_DS_*/*I_DS_*)/*ε*, where *ΔI_DS_* is the drain current change due to strain and *I_DS_* is the current of the unstrained device. Herein, the relative changes of the drain current of five devices of each 6, 8 and 15 nm DNTT layers as a function of applied strain were measured in the strain range of ±0.8%. *ΔI_DS_*/*I_DS_* was observed to significantly increase as the thickness of DNTT decreases from 15 to 6 nm. From those curves, the average values of GFs of DNTT thin films of 6, 8 and 15 nm were calculated to be 42 ± 5, 18 ± 4 and 12 ± 3 and −31 ± 6, −18 ± 4 and −15 ± 4 for applied compressive and tensile strain, respectively. Such a discrepancy in the obtained GF is attributed to the different morphologies of the OSC that largely influence charge transport such as hole mobility and threshold voltage shift [18]. Shifts of *V_th_* have been attributed to a variation of energy for charge accumulation and the number of charge traps in the devices which consequently influence the on-current of the transistors [25]. Other potential effects such as capacitance change of the bilayer gate dielectric under applied strain were also measured in this work. A relative variation of capacitance *ΔC/C* below 2% was recorded for 1% of applied tensile strain and thus considered negligible in light of the large piezoresistive effect of the OSC. Figure 2d plots the changes of the time-resolved drain current at a gate bias voltage of –5 V for three different thicknesses of the active layer (6, 8 and 15 nm) during three cycles of 0.75% of compressive strain and release. The stable stepped current changes demonstrate the reversible and reproducible responsiveness of all sensors. As expected, the largest relative change of the drain current was obtained for a 6 nm thick DNTT-based OFET sensor with a corresponding gauge factor of around 40. 

The morphology of the semiconducting film is strongly correlated to the electrical properties of the organic active layer. To understand the effect of active layer thickness on the piezoresistive sensitivity of DNTT thin film, the morphology of ultrathin active layers with thicknesses of only a few nanometers were investigated from height AFM images of 10 × 10 µm. The observed growth of ultrathin film of DNTT is similar to other high vacuum-deposited organic semiconductors, i.e., pentacene [26,27,28]. As shown in Figure 3, the images revealed the direct effect of thin film thickness on their morphology and ability to transport charge carriers along the transistor channel. The morphology of the 5 nm thick DNTT thin film consists of grains too far away from each other to allow charge transport across the transistor channel. On the contrary, an AFM scan of 15 nm revealed a uniform thin film of DNTT with the largest on-state *I_DS_* values due to a very high number of (quasi-3D) critical electrical percolation paths. For intermediate thicknesses (i.e., 6 and 8 nm), height AFM images showed two similar morphologies where DNTT grains get closer to each other and create only a limited number of (quasi-2D) critical electrical percolation paths to allow charge transport, which is highly sensitive to strain. In fact, the average grain diameter was observed to increase with the thickness of the OSC layer. The average grain sizes of 5, 6 and 8 nm thick films were measured to be 318, 356 and 429 nm, respectively.

In summary, we have demonstrated the functionality of piezoresistive strain sensors based on flexible DNTT OFETs. The electrical properties of DNTT were shown to be highly dependent on the semiconducting film morphology that is affected by film thickness. More specifically, the piezoresistive effect of DNTT was explained to be a strain-induced morphological change of the active layer, notably the distance between adjacent DNTT grains altering hole transport along the transistor channel. The strain sensitivity of the sensors was found to be dependent on the OSC thickness affecting the average grain size and hence substrate coverage. We demonstrated that the GF can be tuned by adjusting the OSC thickness close to the percolation threshold. An optimized active layer of 6 nm, i.e., not continuously covered, presented a GF of over 40 under compressive strain whereas an active layer thicker than 15 nm, i.e., full coverage, exhibits a GF of 12. Identification of such a critical thickness in high performance and stable DNTT OFET sensors also opens up further applications, as it contains abundant sites for various chemical or biological analyte binding for efficient current modulation. 

## Figures and Tables

**Figure 1:**
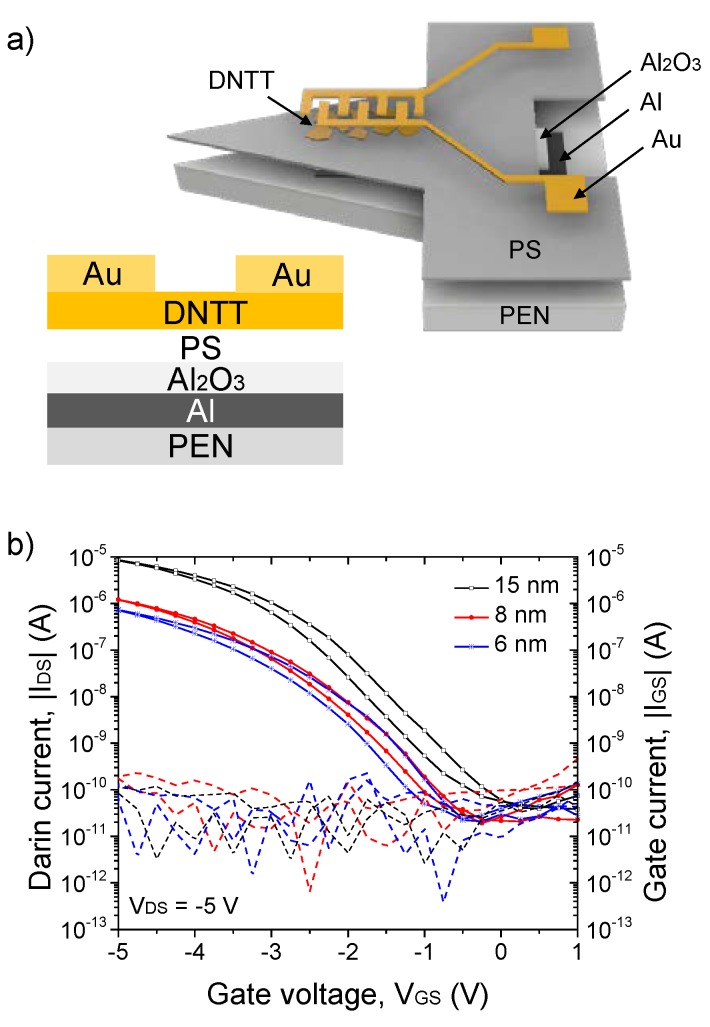
(**a**) Schematic side view of the CantiFET device and its corresponding cross section and (**b**) transfer characteristics of CantiFET with 6, 8 and 15 nm of dinaphtho [2,3-b:2,3-f] thieno [3,2–b] thiophene (DNTT) active layer (solid lines) and their corresponding leakage current (dash lines).

**Figure 2:**
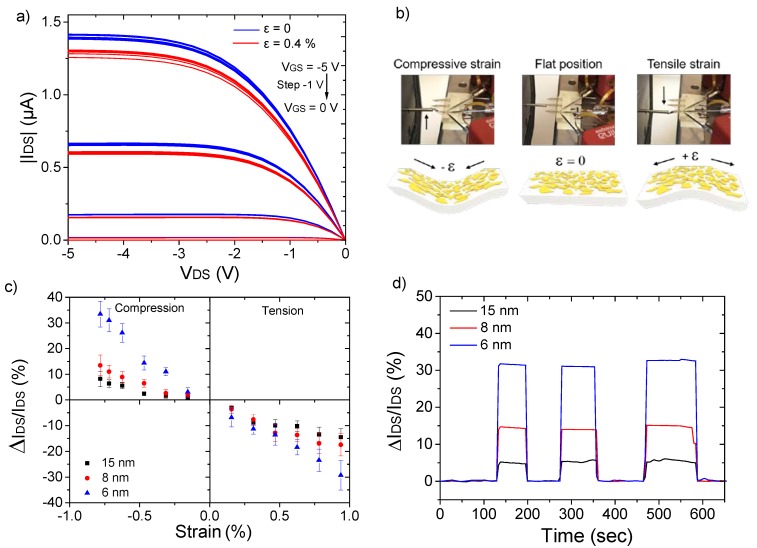
(**a**) Output curves of an 8 nm thick DNTT-based organic field-effect transistor (OFET) sensors under 0.4% of tensile strain, (**b**) optical images of the sensors subjected to compressive strain, in its flat position and under tensile strain with their corresponding schematics illustrating the modification of the active layer morphology under applied strain, (**c**) relative change of drain current of 6, 8 and 15 nm thick sensors under applied compressive and tensile strain at *V_DS_* = −5 V and (**d**) relative change of drain current of 6, 8 and 15 nm thick active layer when subjected to 0.75% of compressive strain at *V_DS_* = −5 V.

**Figure 3:**
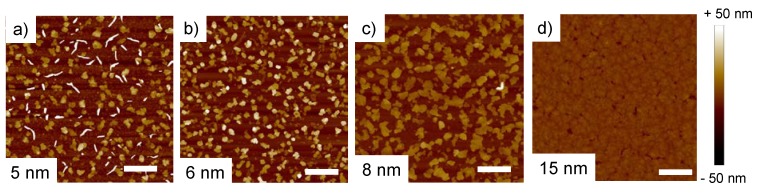
AFM height images of DNTT thin films of thicknesses of (**a**) 5 nm, (**b**) 6 nm, (**c**) 8 nm and (**d**) 15 nm. Scale bar: 2 µm.
